# Multiple gene movements into and out of haploid sex chromosomes

**DOI:** 10.1186/s13059-017-1201-7

**Published:** 2017-06-08

**Authors:** Agnieszka P. Lipinska, Nicholas R. T. Toda, Svenja Heesch, Akira F. Peters, J. Mark Cock, Susana M. Coelho

**Affiliations:** 1 Sorbonne Université, UPMC Univ Paris 06, CNRS, Algal Genetics Group, UMR 8227, Integrative Biology of Marine Models, Station Biologique de Roscoff, CS 90074, F-29688 Roscoff, France; 2 Bezhin Rosko, 29250 Santec, France

**Keywords:** Sex chromosomes, Brown algae, Gene movement, Gene transposition

## Abstract

**Background:**

Long-term evolution of sex chromosomes is a dynamic process shaped by gene gain and gene loss. Sex chromosome gene traffic has been studied in XY and ZW systems but no detailed analyses have been carried out for haploid phase UV sex chromosomes. Here, we explore sex-specific sequences of seven brown algal species to understand the dynamics of the sex-determining region (SDR) gene content across 100 million years of evolution.

**Results:**

A core set of sex-linked genes is conserved across all the species investigated, but we also identify modifications of both the U and the V SDRs that occurred in a lineage-specific fashion. These modifications involve gene loss, gene gain and relocation of genes from the SDR to autosomes. Evolutionary analyses suggest that the SDR genes are evolving rapidly and that this is due to relaxed purifying selection. Expression analysis indicates that genes that were acquired from the autosomes have been retained in the SDR because they confer a sex-specific role in reproduction. By examining retroposed genes in *Saccharina japonica*, we demonstrate that UV sex chromosomes have generated a disproportionate number of functional orphan retrogenes compared with autosomes. Movement of genes out of the UV sex chromosome could be a means to compensate for gene loss from the non-recombining region, as has been suggested for Y-derived retrogenes in XY sexual systems.

**Conclusion:**

This study provides the first analysis of gene traffic in a haploid UV system and identifies several features of general relevance to the evolution of sex chromosomes.

**Electronic supplementary material:**

The online version of this article (doi:10.1186/s13059-017-1201-7) contains supplementary material, which is available to authorized users.

## Background

Gene loss and gene gain events have been shown to play an important role in the evolution of XY and ZW sex chromosomes in several different metazoan lineages. Species with XY chromosomes have a significant excess of genes moving out of the X chromosome compared with autosomes, and these genes tend to acquire male-biased or male-specific expression, suggesting they play a role in male reproduction [1–4]. The major evolutionary mechanisms that have been put forward to explain out-of-X gene movements are meiotic sex chromosome inactivation (MSCI) [1, 5], sexual antagonism [6, 7], dosage compensation [8, 9] and meiotic drive [10]. In organisms with female heterogamety (ZW systems), an equivalent process is observed, involving gene relocation out of the Z chromosome to autosomes and acquisition of female-biased (ovary-biased) expression patterns [11]. X and Z chromosomes are not only characterised by loss of genes but they have also experienced recurrent and convergent gain of individual genes or genomic regions, most of which have male beneficial roles [12].

Similarly, the evolution of the sex chromosome that is present in only one sex in heterogametic systems (Y or W) has been shaped by both loss and gain of genes. The therian X and Y and the avian Z and W diverged ~160 million and ~130 million years (MY) ago, respectively. Subsequently, the Y/W chromosomes lost most of the genes that were initially present on the autosomes from which they originated. For primates and *Drosophila*, the genes that remain on the Y tend to have male-specific functions [13], but this is not the case for the genes that have remained on the female-specific W in birds and these W chromosomes do not appear to have a role in determining female fertility [14]. A variety of evolutionary models have been proposed to explain the degeneration of the Y across all organisms studied so far [13]. A common feature of these models is that the efficacy of natural selection is strongly reduced on a non-recombining chromosome. Interestingly, genes have also moved into the Y chromosome in some organisms. For instance, gene gain has played a prominent role in the evolution of the *Drosophila melanogaster* Y [15]. Genes that transpose onto the Y from an autosomal location tend to acquire male functions [5, 16, 17]. Dynamic changes in the gene content of plant Y chromosomes, including loss of genes and gain of genes with a role in male reproduction, have also been documented [6, 18, 19].

Studies of the dynamics of gene movement into and out of the sex chromosomes have focused on model organisms with XY or ZW sex chromosomes, and we know virtually nothing about the dynamics of gene gain/loss in a third type of sex chromosome system that exists in multicellular eukaryotes, the UV sex chromosomes [20]. In UV systems, the sex of the individual is determined and expressed during the haploid stage of the life cycle depending on whether the spore receives a U or a V chromosome following meiosis. Although UV systems share many common features with XY/ZW systems, they also have several differences and these have important evolutionary and genomic implications. As with XY or ZW systems, UV systems are expected to evolve suppression of recombination provided there is a difference in strength and/or direction of selection between the sexes [21]. Differences in selection are expected to result in tighter linkage of female-beneficial alleles to the U chromosome and male-beneficial alleles to the V chromosome, respectively, and this will eventually lead to expansion of the non-recombining region. Also as in XY/ZW systems, loss of recombination and accumulation of deleterious alleles by drift and background selection (e.g., [22]) can favour the appearance and spread of functional copies of sex chromosome genes on the autosomes, followed by their loss from the U- and V-specific regions [21]. In contrast to XY and ZW systems, however, degeneration by accumulation of deleterious mutations on U and V chromosomes should affect the two chromosomes symmetrically and to a lower degree than it affects W or Y, because they do not experience as marked a reduction in the effective population size (one-half for U or V compared to autosomes but one-quarter for Y or W) and because the U and V are exposed to haploid selection [21, 23]. For these reasons, sex chromosome degeneration is expected to occur more slowly in UV compared to XY/ZW systems and the former should have a larger fraction of long-standing functional genes. Alternative alleles of genes on the U and V that are subject to balancing selection, sexually antagonistic selection and/or ploidy-antagonistic selection are expected to be rapidly fixed, contributing to a high level of differentiation [21]. As a result of these differences between XY/ZW and UV systems, comparative analyses of the two classes of system can provide important insights into the evolutionary processes underlying sex chromosome evolution.

Brown algae are a group of predominantly marine organisms that are of particular interest from an evolutionary point of view because they represent one of the five major lineages that evolved complex multicellularity and because they are very distantly related to land plants and animals (more than one billion years of independent evolution). Brown algae display a remarkable diversity of morphological complexity and include some of the largest organisms on earth. The Ectocarpales and the Laminariales (kelps) are two major brown algae orders that diverged from each other about 80–110 MY ago [24, 25]. They exhibit a high level of diversity in terms of their life cycles, degree of sexual dimorphism and the morphological complexity of the sporophyte and gametophyte generations [26]. The members of both orders have haploid–diploid life cycles and UV sex chromosome systems [27]. The U and V sex chromosomes of the brown algal model *Ectocarpus* have been recently characterised, and there is evidence that the U and V sex chromosomes of the Ectocarpales and the Laminariales have a common origin [27, 28].

In this study, we have used the recently published genome sequence of the kelp *Saccharina japonica* [29] together with sex-specific genome and transcriptome data for six additional brown algal species to investigate the dynamics of U- and V-linked gene content across 100 MY of evolution with a focus on the mechanisms underlying the movement of genes into and out of the sex-determining region.

## Results

### Conservation of sex-determining region gene content among brown algae

Sequence analysis of the U and V sex chromosomes of *Ectocarpus* sp. showed that the male haplotype of the SDR contains 17 protein-coding genes and two pseudogenes, whereas the female haplotype consists of 15 protein-coding genes and seven pseudogenes (Additional file 1: Table S1) [28]. Nine of the female protein-coding genes and two of the pseudogenes are homologous to male SDR sequences (i.e., are “gametologues”), whereas the rest of the genes are “sex-limited”, i.e., genes that are exclusively on the male or female SDR, without a homologous sequence in the other haplotype (Additional file 1: Table S1).

We performed a systematic search for orthologues of *Ectocarpus* sp. SDR genes in the genomes of six brown algal species (*Ectocarpus siliculosus*, *Ectocarpus fasciculatus*, *Scytosiphon lomentaria*, *S. japonica*, *Macrocystis pyrifera* and *Undaria pinnatifida*, which present increasing evolutionary distances from the reference species *Ectocarpus* sp.; Additional file 1: Table S2). For this, whole genome sequences were generated for males and females of four of the species, and we used publicly available sequences for *U. pinnatifida* (Additional file 1: Table S2). The sequence of the *S. japonica* genome has been published [29]. The genomic locations of orthologues of the *Ectocarpus* sp. SDR genes (sex-linked versus autosomal) were assessed in these six species using bioinformatic approaches and experimentally validated using PCR (see the “Methods” section for details). Note that we excluded pseudogenes from the analysis in most cases, the only exceptions being two female pseudogenes that belong to gametologue pairs, which were included to provide complete information about the fate of these gametologue pairs across species (Additional file 1: Table S1).

**Fig. 1:**
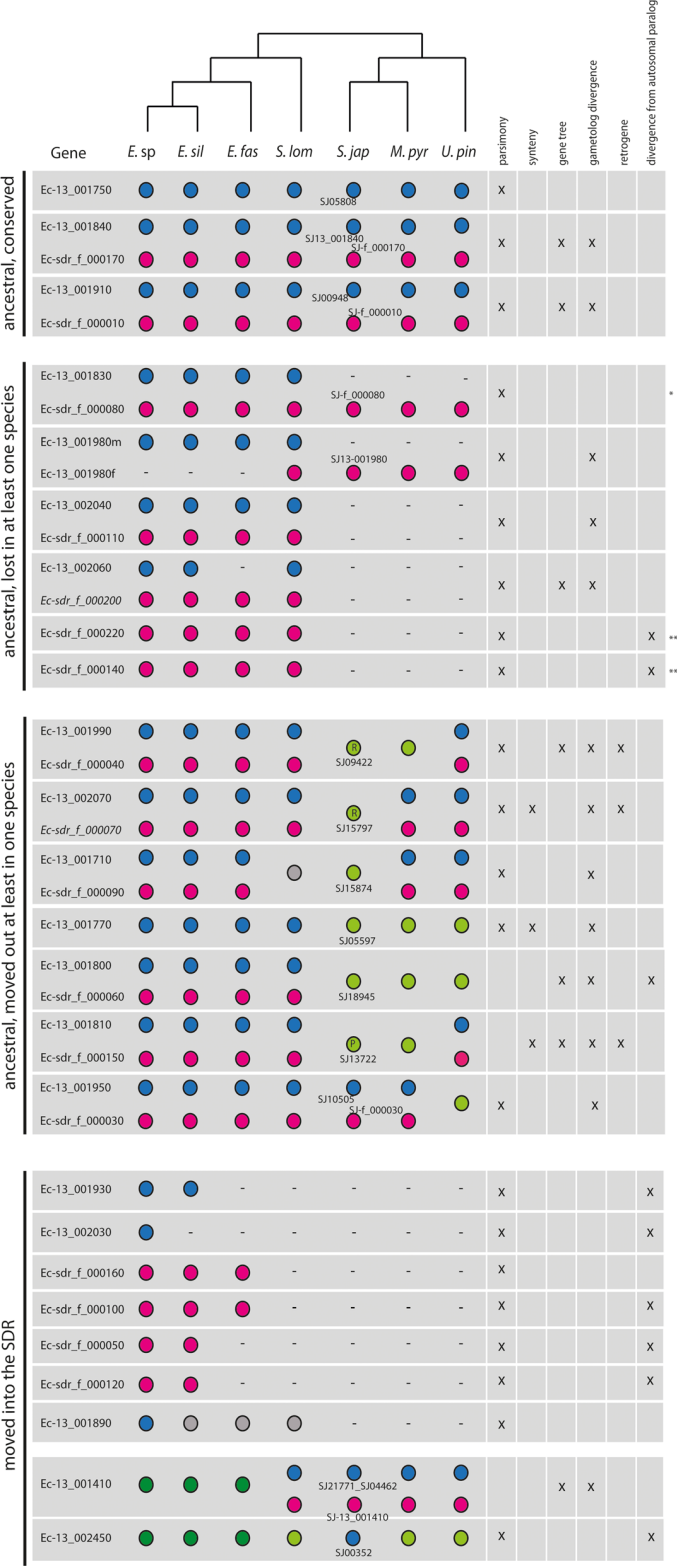
Presence, location and characteristics of sex-linked, protein-coding genes and their pseudoautosomal region (PAR) and autosomal orthologs in seven brown algal species. Gene names are based on the *Ectocarpus* sp. annotation. For genes that were not annotated in the *S. japonica* reference genome [29], we used an abbreviation based on the *Ectocarpus* sp. gene (for instance, SJ-13_001840 corresponds to the ortholog of Ec-13_001840). Pseudogenes that are part of gametologue pairs are represented in italics. The presence of a male or female sex-linked gene in a given species is indicated by a *blue* or *pink circle*, respectively. Absence of a gene is indicated by a *minus sign. Dark green circles* indicate genes located in the PAR, *light green circles* represent either a PAR or an autosomal location. *Grey circles* indicate that the genomic location of a gene is unclear. *Green circles* with an *R* indicate retrogenes. The *green circle* with a *P* indicates a pseudoretrogene. The deduced history of each group of genes is shown to the *left* and the arguments on which the decision was based for each gene or gametologue pair are indicated with a *cross* to the *right*. *Blast results were ambiguous for this gene so orthology was determined by phylogeny; **we cannot totally exclude a scenario where these genes entered the SDR after the split of the Laminariales and Ectocarpales lineages. *E.* sp, *Ectocarpus* sp.; *E. sil*, *E. siliculosus*; *E. fasc*, *E. fasciculatus*; *S. lom*, *S. lomentaria*; *S. jap*, *S. japonica*; *M. pyr*, *M. pyrifera* ; *U. pin*, *U. pinnatifida*

We found putative orthologues for all except one of the *Ectocarpus* sp. male and female SDR genes (33 of the 34 genes) in at least one of the two other *Ectocarpus* species, *E. siliculosus* and *E. fasciculatus* (Fig. 1). In general, genes that were members of gametologous pairs in *Ectocarpus* sp. were also detected as sex-linked, gametologous pairs in *E. siliculosus* and *E. fasciculatus*. In contrast, about half (5 out of 12) of the *Ectocarpus* sp. male- or female-limited genes were either not sex-linked or were totally absent from the genomes of at least one of the two *Ectocarpus* species, most often *E. fasciculatus* (Fig. 1).

Comparison with *S. lomentaria*, a more distantly related brown alga belonging to a different family within the Ectocarpales, and with three kelp species, *S. japonica*, *M. pyrifera* and *U. pinnatifida*, indicated that the gene content of the SDR is substantially more dynamic at large evolutionary scales. Only just over half of the *Ectocarpus* sp. SDR genes (59%, 20 of the 34 SDR genes) had orthologues in all six of the other brown algal species, and only seven of these 20 genes were sex-linked in all seven species. The conserved sex-linked genes included two gametologue pairs (Ec-13_001840/Ec-sdr_f_000170, Ec-13_001910/Ec-sdr_f_000010), two sex-limited genes (Ec-13_001750, Ec13_001980) and one female gametologue (Ec-sdr_f_000080). The only gene that was consistently male-limited in all the species studied was the HMG domain protein-encoding gene Ec-13_001750, which has been proposed as a candidate for the male sex-determining gene [28].

### Gene content of the ancestral SDR

The presence of several shared gametologue pairs in the SDR regions of all the brown algal species studied here strongly indicated that these genes were already sex-linked in the last common ancestor of kelps and Ectocarpales, 80–110 MY ago. The extremely high levels of pairwise divergence between gametologues, measured as dS, were also consistent with an ancient origin for these gametologue pairs. Values of synonymous substitution (dS) across species were relatively uniform and reached saturation in most instances (Additional file 1: Table S3). Maximum likelihood trees were built for genes that were present in more than four species (Additional file 2: Figure S1). Overall, in trees built for genes that belonged to gametologue pairs the sequences tended to cluster by sex and not by species (Additional file 2: Figure S1), consistent with suppression of recombination between U and V chromosomes before the divergence of the kelps and the Ectocarpales and confirming previous results using a smaller sample size [27, 28].

**Fig. 2:**
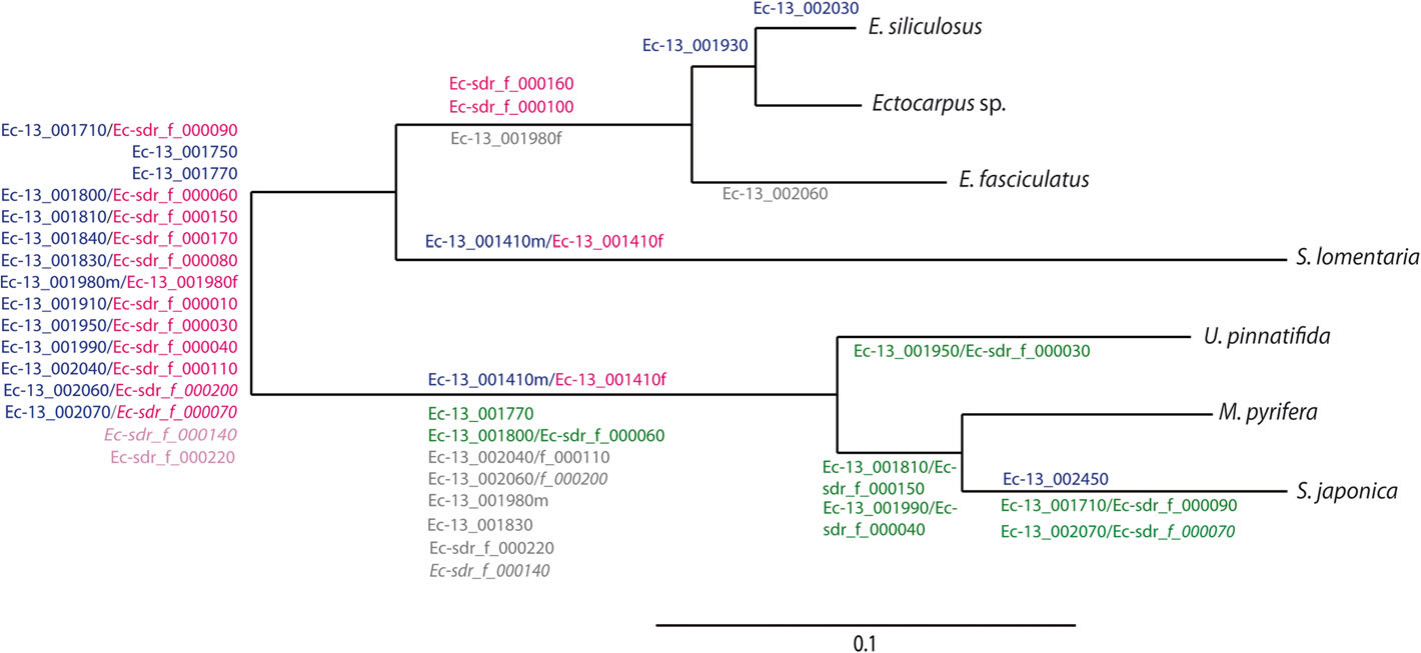
Overview of the evolutionary history of gene gain and gene loss from brown algal SDRs. The maximum likelihood phylogenetic tree based on cox1–cox3 sequences (PhyML, HKY85 model, 500 bootstraps) represents the evolutionary relationships among species analysed in the current study. Branch lengths are proportional to lineage divergence times. Genes at the base of the tree are predicted to be ancestral in the SDR; the estimated timing of proposed gene gain and gene loss events is indicated by names placed above or below specific branches of the tree, respectively. Gene names, which are indicated based on the *Ectocarpus* sp. orthologue, are colour-coded to indicate the current chromosomal location—male SDR (*blue*), female SDR (*pink*), autosomal (*green*)—or to indicate gene loss (*grey*). The two genes for which SDR ancestry is unclear are marked in *light pink*

Comparative analysis of sex-linked genes across the seven brown algal species allowed us to conservatively infer that the U and V SDRs of the common ancestor of kelps and *Ectocarpales* were composed of at least 26 genes, including 12 pairs of gametologues and two male-limited genes (Fig. 2).

### Molecular evolution of SDR genes

We next investigated the consequences of sex linkage on the molecular evolution of genes that have been residing in the SDR of the different lineages, specifically focusing on *Ectocarpus* sp. and *S. japonica* because these two genomes have the highest quality annotations. In XY and ZW sex chromosome systems, the efficacy of purifying selection is markedly reduced for Y/W linked genes relative to genes on other chromosomes due to suppression of recombination and reduced effective population size [30]. In UV sex chromosome systems, selection is also expected to be less efficient within the non-recombining SDR. We therefore compared the rates of evolution, as measured by the ratio of non-synonymous over synonymous substitutions (dN/dS), of SDR genes with those of genes located in recombining regions of the genome.

**Fig. 3:**
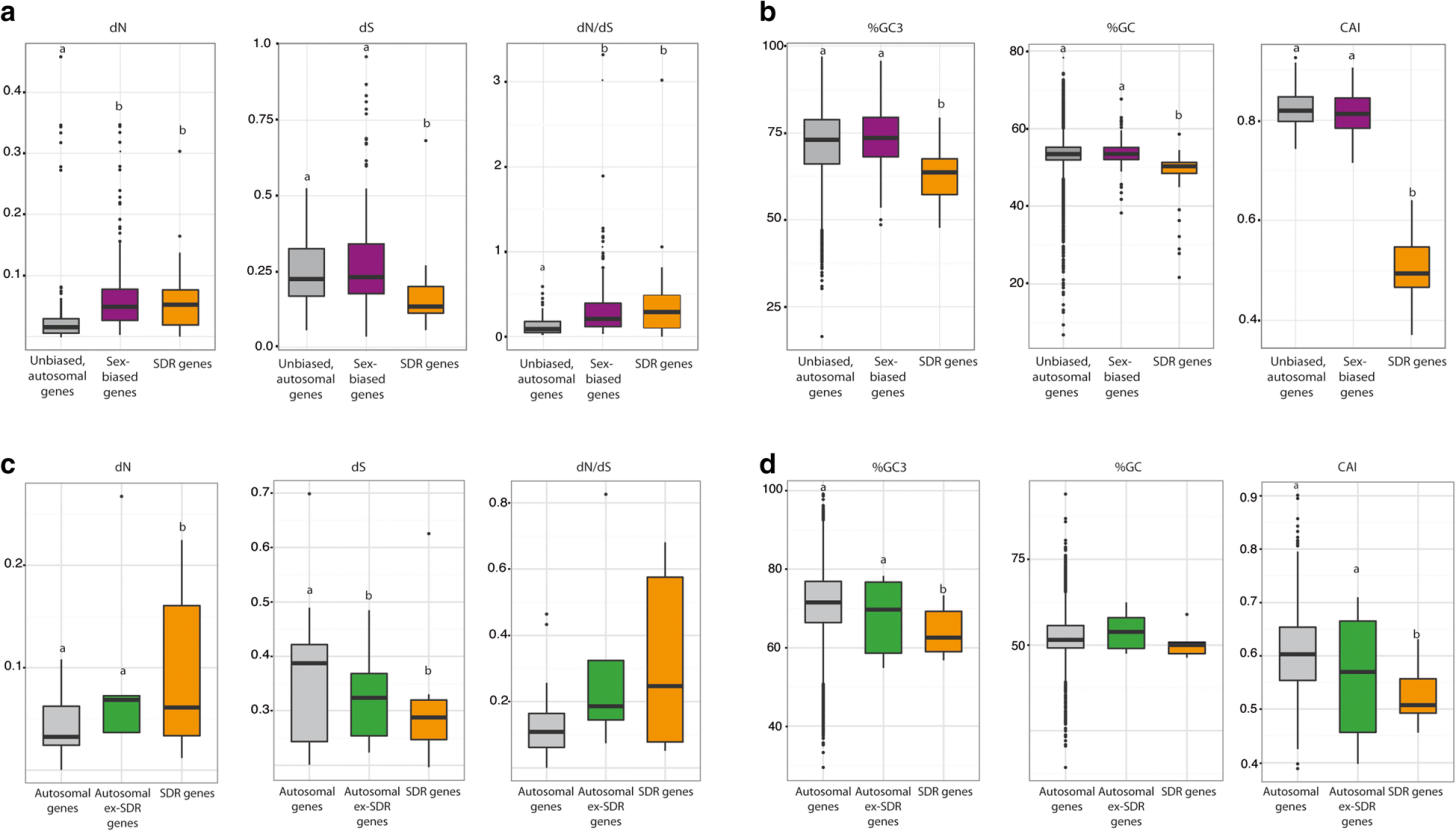
Evolutionary features of sex-linked (SDR) genes in *Ectocarpus* sp. and in the kelp *S. japonica*. **a** dN, dS and dN/dS for *Ectocarpus* sp. SDR genes compared with sex-biased and autosomal genes. **b** GC3, GC and codon usage bias (analysed as codon adaptive index (*CAI*)) for *Ectocarpus* sp. genes compared with sex-biased and autosomal genes. **c** dN, dS and dN/dS for *S. japonica* SDR genes compared with autosomal genes and genes that have moved out of the SDR (ex-SDR genes). **d** GC3, GC and codon usage bias (CAI) for *S. japonica* SDR genes compared with autosomal genes and genes that have moved out of the SDR (ex-SDR genes)

Overall, SDR genes exhibited higher dN/dS values than autosomal genes (Wilcoxon test, *p* = 0.00019) due to elevated dN (Wilcoxon test, *p* = 0.0036) and reduced dS values (Wilcoxon test, *p* = 0.00053) (Fig. 3a; Additional file 1: Table S4). Evolutionary rates can be affected by gene expression pattern, in particular breadth of expression [31, 32]. We therefore also compared the dN/dS ratios of sex-linked genes with those of sex-biased genes, because the latter exhibit narrow (sex-specific) expression patterns similar to those observed for SDR genes [33]. Evolutionary rates for SDR genes were comparable to those for sex-biased autosomal genes (Wilcoxon test, *p* = 0.19501) but dS values were significantly lower for SDR genes (Wilcoxon test, *p* = 0.00017) (Fig. 3a).

High dN/dS values may be indicative of either reduced efficacy of purifying selection or positive selection. The majority of genes on animal Y chromosomes evolve under relaxed purifying selection [34] but there is evidence that positive selection acts on some of these loci [35–37]. Similarly, the faster rate of evolution of sex-biased genes in *Ectocarpus* sp. has been partially attributed to adaptive processes [33]. However, a suite of evolutionary tests (Additional file 1: Table S5) provided no evidence that the genes in the U and V SDR regions were evolving under positive selection.

SDR genes exhibited less codon usage bias (CUB; Wilcoxon test *p* < 2.2e^–16^; see also [28]) and reduced GC3 (Wilcoxon test, *p* = 7.2e^–08^) compared with autosomal genes, suggesting relaxed purifying selection acting on the SDR (Fig. 3b). GC levels were also lower in SDR genes (Wilcoxon test *p* < 2e^–16^), which may be due to AT mutational bias and accumulation of GC-poor transposons in the introns of SDR genes or to selective GC substitutions and GC-biased gene conversion acting on autosomal genes [38, 39].

CUB may be influenced by gene expression level, and indeed a positive correlation between CUB and gene expression has been reported previously for *Ectocarpus* sp. [33]. Analysis of RNA-seq data using the highest expression level for a given gene across several life stages [33, 40] detected no significant differences between SDR and autosomal genes (Wilcoxon test *p* = 0.903, median log_2_TPM = 5.26 (95% CI 5.23, 5.30) and 5.25 (95% CI 4.63, 5.87) for autosomal and SDR genes, respectively), indicating that neither the decreased CUB of SDR genes nor their faster evolutionary rates were caused by decreased expression levels.

Compositional features of SDR genes of the kelp *S. japonica* resembled those of *Ectocarpus* sp. Both the codon adaptive index (CAI) and the GC content of *S. japonica* sex-linked genes were significantly lower than those of autosomal genes (Mann–Whitney U test, *p* < 2.2e-16; Fig. 3; Additional file 1: Table S4), consistent with reduced efficacy of purifying selection acting on the SDR. The *S. japonica* SDR genes also showed accelerated evolutionary rates, as measured by pairwise dN/dS, compared with autosomal genes (Additional file 1: Table S4; Fig. 3c). Interestingly, the majority of the genes that have been relocated to autosomes in *S. japonica* also exhibited higher dN/dS and lower GC and CAI (Additional file 1: Table S4; Fig. 3c, d).

Taken together, our results indicate accelerated evolution of SDR genes in both the kelp and the *Ectocarpus* lineages. The faster evolutionary rates were likely due to decreased efficacy of purifying selection since we found no evidence for positive selection acting on any of the SDR genes.

### A candidate sex-determining gene among the core set of genes present in the ancestral UV sex-determining region

Three genes were found to be V-linked in all the brown algae studied here (Ec-13_001750, Ec-13_001840 and Ec-13_001910; Fig. 1), making them potential candidates for the male sex-determining factor(s). Among them, Ec-13_001750 is a particularly strong candidate. First, this is the only gene that is limited to the male SDR across all seven species; the other two have gametologues. Second, Ec-13_001750 exhibits a pattern of expression in *Ectocarpus* sp. that is consistent with a role in sex determination, being strongly upregulated at fertility during the production of male gametes [28] (Additional file 2: Figure S2). Finally, this gene is predicted to encode a HMG domain-containing protein and therefore belongs to the same class of genes as the sex-determining genes in several animal and fungal species [41, 42].

### Gene traffic in and out of the SDR

We assessed gene movement in/out of the SDR on a gene-to-gene basis based on a number of criteria: Dollo parsimony applied to comparative genomics, local chromosomal gene synteny, configurations of maximum-likelihood gene trees, divergence between gametologues, presence of a retrocopy, and degree of divergence between SDR genes and their autosomal paralogs.

#### Genes that have moved out of the SDR

**Fig. 4:**
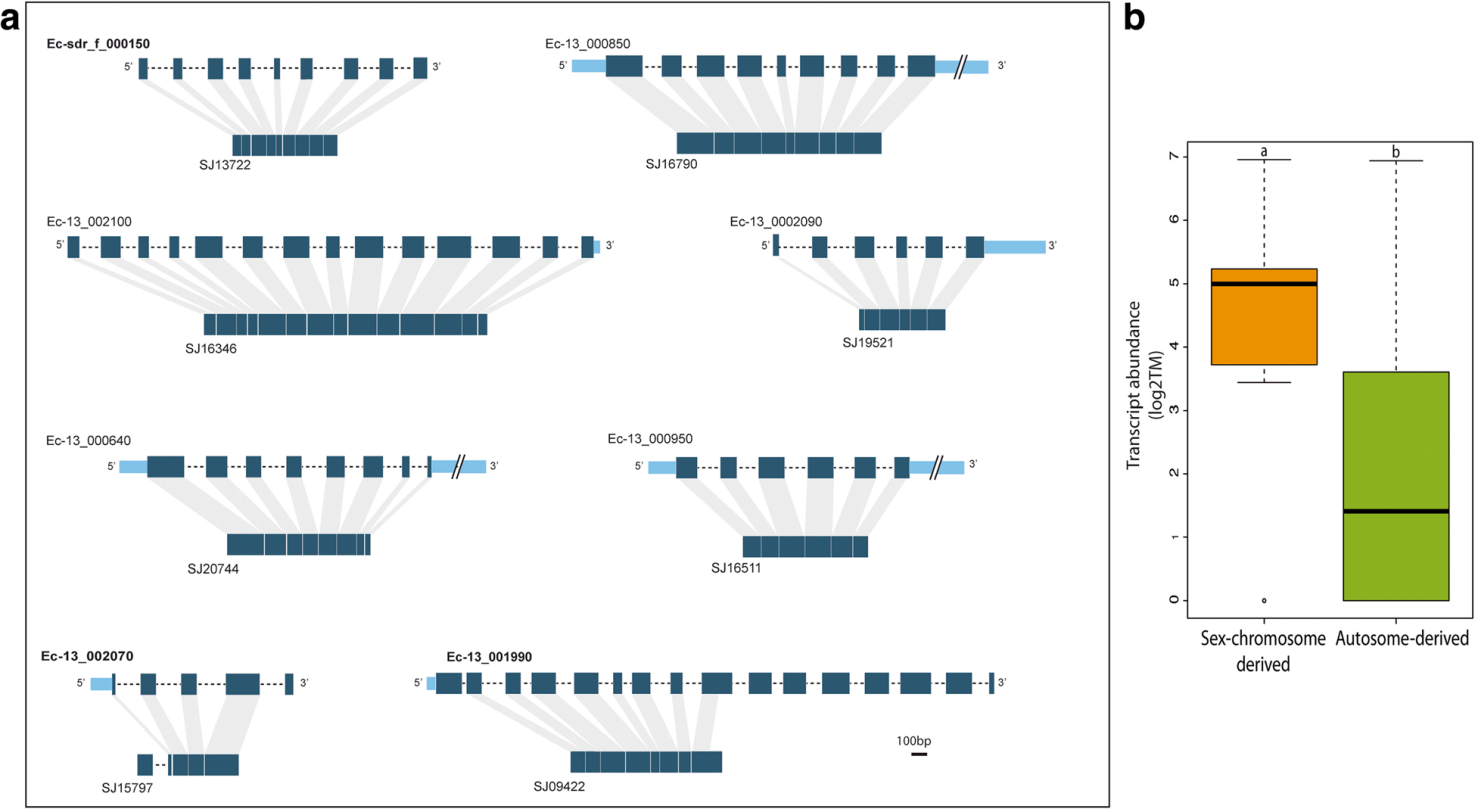
Retrogenes in *S. japonica*. **a** Schematic alignments of *Ectocarpus* sp. intron-rich sex-chromosome genes (*top*) and intronless, autosomal orthologs in *S. japonica* (*bottom*). Genes that are located on the *Ectocarpus* sp. SDR are highlighted in bold. Exons, *dark blue*; UTRs, *light blue*; introns, *dotted lines* (introns are not shown to scale). **b** Expression of *S. japonica* intronless retrogenes whose orthologs in *Ectocarpus* sp. are either located on the sex chromosome (*Sex-chromosome-derived*) or autosomal (*Autosome-derived*)

**Fig. 5:**
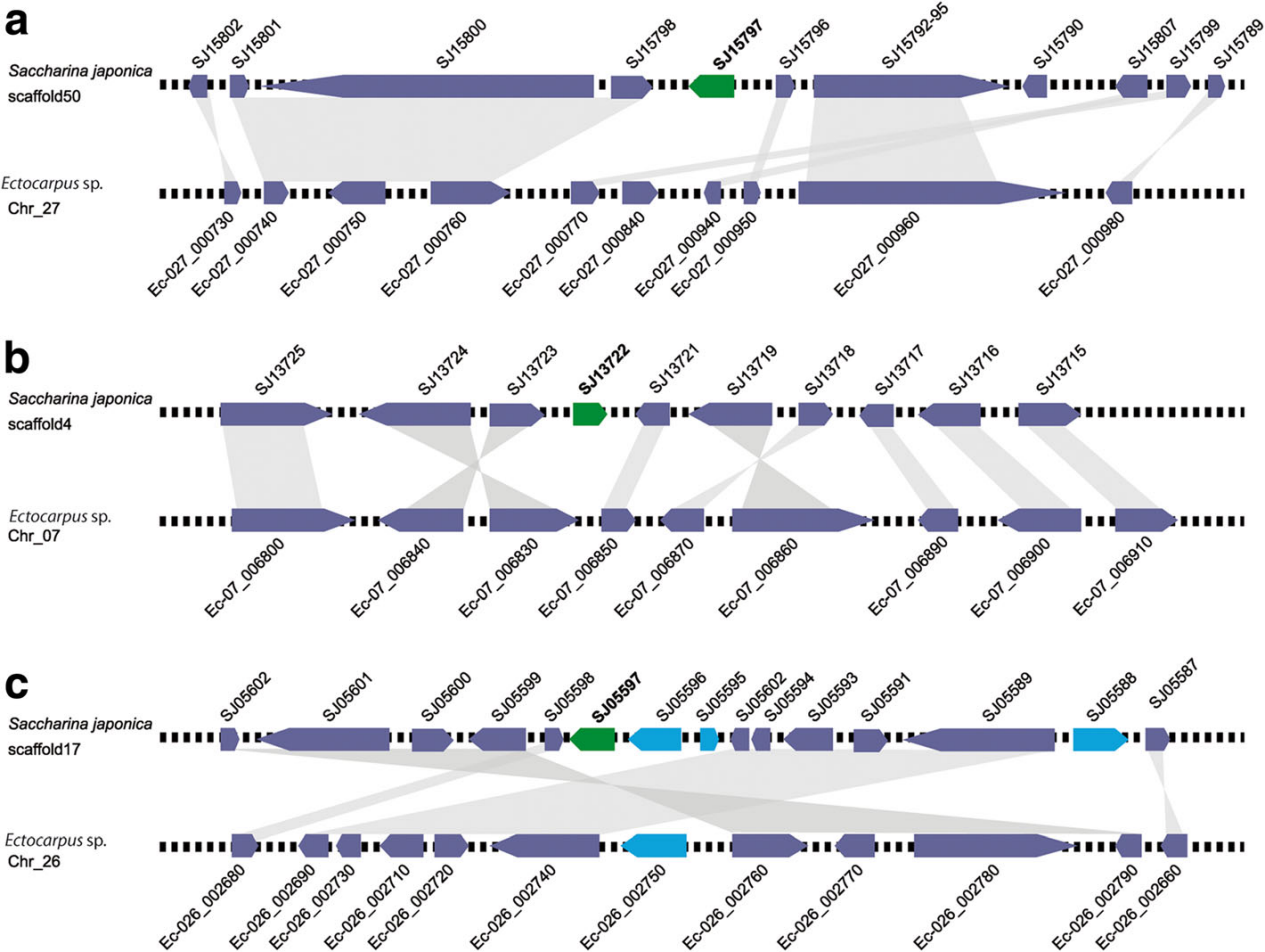
Comparisons between the genomic regions surrounding *S. japonica* orthologues SJ15797 (**a**), SJ13722 (**b**) and SJ05597 (**c**) of three *Ectocarpus* sp. SDR genes (Ec-13_002070, Ec-13_001810 and Ec-13_001770, respectively) and the equivalent, syntenic regions in the *Ectocarpus* sp. genome. *Green boxes* indicate the *S. japonica* orthologues of the three *Ectocarpus* sp. SDR genes, *violet boxes* indicate syntenic orthologues, *blue boxes* indicate genes without syntenic orthologues. *Chr* chromosome

We found seven examples of genes that have moved out of the ancestral SDR to other genomic regions. The evidence for SDR-to-autosome gene movement was very compelling for some genes. For instance, in three cases where genes have relocated to autosomes in kelps after separation of the *Ectocarpus* and kelp lineages (Ec-13_002070/SJ15797, Ec-13_001810/SJ13722 and Ec-13_001990/SJ09422), there was evidence that the movement involved an RNA-based, retrotransposition mechanism and the intron loss associated with the transfer provided strong evidence for the parent–child relationship and therefore the direction of movement (Figs. 1 and 4a). Similarly, analysis of the genomic context around individual genes (synteny) provided additional support for the movement of two of the above genes (Ec-13_002070/SJ15797 and Ec-13_001810/SJ13722) and provided strong evidence for an additional gene (Ec-13_001770/SJ05597) having moved out of the SDR in the kelp lineage (Fig. 5). The *S. japonica* retrogenes SJ05797 and SJ13722 are flanked on both sides by genes whose orthologues are clustered in a single autosomal region of the *Ectocarpus* sp. genome, providing convincing evidence that SJ15797 and SJ13722 have been inserted in these regions as single genes through retrotransposition (Fig. 5a, b). The relocation of SJ05597 appears to have involved the movement of a genomic segment consisting of at least three genes (Fig. 5c). Movement of SJ15874 out of the SDR is supported by the high divergence level of the gametologues across all the species as well as by the presence of the two gametologues in the other two kelp species, strongly arguing that this is an ancestral gametologue pair.

#### Gene loss

Orthologues of some of the genes that were presumed to have been present in the ancestral SDR based either on gametologue divergence (Ec-13_001830, Ec-13_001980, Ec-13_002040, Ec-13_002060) or on a high level of divergence from the most similar autosomal gene (Ec-sdr_f_000220, Ec-sdr_f_000140) could not be found in the genomes of at least one of the brown algal species, indicating these genes may have been lost. For example, the male gametologue Ec-13_002060 appears to have been lost from the *E. fasciculatus* genome and no orthologues for either the male or the female gametologue (Ec-13_002060 nor Ec-sdr_f_000200) were found in kelps. A similar case of gene loss is represented by the pair Ec-13_002040/Ec-sdr_f_000110, where neither gametologue was found in the kelp genomes. Interestingly, Ec-13_001980 belongs to a gametologue pair in *S. lomentaria* whereas orthologs of this gene in all other species are limited to only one sex. The three *Ectocarpus* species have the male gametologue and the three kelps have the female gametologue. The most parsimonious scenario would thus be that the *Ectocarpus* species lost the female gametologue whereas the kelp species lost the male. Further support for this hypothesis includes the high level of divergence between the two gametologues in *S. lomentaria*, suggesting that these genes have not recombined for a long period of time (Additional file 1: Table S3; Fig. 1).

#### Genes that have moved into the SDR

We found evidence for at least nine genes having moved into the SDR (Fig. 1). For eight of these genes (Ec-13_001930, Ec-13_002030, Ec-sdr_f_000160, Ec-sdr_f_000100, Ec-sdr_f_000050, Ec-sdr_f_000120 and Ec-13_001890 and Ec-13_002450) movement was inferred based on parsimony arguments (i.e., it was more likely that there had been one gene movement event into the SDR than multiple events involving gene deletion or movement out of the SDR) and on the presence of a closely related autosomal homologue in the *Ectocarpus* sp. genome, which could potentially correspond to the parental gene. Autosomal orthologues of two of these genes (Ec-13_001930 and Ec-13_001890) were found in one or more species outside the genus *Ectocarpus*, suggesting that at least these may have moved into the SDR from an autosomal location (Additional file 1: Table S6). Interestingly, for two genes (Ec-13_001930 and Ec-sdr_f_000120) the autosomal homologue is on the pseudoautosomal region (PAR), suggesting that these genes have moved into the SDR from the PAR via a recent gene duplication and translocation event.

An alternative mechanism that could lead to movement of genes into the SDR is expansion of the SDR to subsume genomic regions that originally corresponded to the PAR. We found evidence that this had occurred for at least two *Ectocarpus* sp. PAR genes, Ec-13_001410 and Ec-13_002450. Male and female sex-linked orthologues (gametologues) of Ec-13_001410 were found in *S. lomentaria* and in the three kelps, suggesting that the region containing Ec-13_001410 had been subsumed into the SDR twice, once in the kelps and once in the *S. lomentaria* lineage. The SDR therefore appears to have independently expanded in both of these lineages. This scenario is supported by the configuration of the phylogenetic tree for Ec-13_001410 (Additional file 2: Figure S1). Moreover, the *S. japonica* orthologue of Ec-13_001410 (SJ21771_SJ04462) exhibited significantly higher dN/dS and dN compared to autosomal genes in kelps, consistent with an SDR location (Additional file 1: Table S4). The *S. japonica* orthologue of Ec-13_002450 (SJ00352) is sex-linked, suggesting that this region of the ancestral PAR was similarly assimilated into the SDR specifically in this kelp (the *U. pinnatifida* and *M. pyrifera* orthologues of this gene are not sex-linked). SJ00352 showed lower dN/dS and dN than the autosomal average (Additional file 1: Table S4), which further indicates that it was only recently incorporated into the SDR of *S. japonica*.

In summary, although there is evidence for a conserved set of sex-linked genes shared across kelps and Ectocarpales, marked rearrangements of the SDR occurred in a lineage-specific fashion, involving gene loss, gene gain from autosomes and relocation from the SDR to autosomes.

### Gene movement and expression patterns

Models on the evolution of sex chromosomes predict that genes advantageous for one sex and detrimental for the other (sex antagonistic) accumulate in the chromosome specific to the sex they are good for (Y or W, but also U or V) [43]. For instance, many genes on the Y chromosomes of *Drosophila* and mammals have male-specific functions (reviewed in [13]). Therefore, we investigated if genes that entered the brown algal U- and V-specific regions presented a pattern of expression consistent with a role in reproduction in the corresponding sex. We focused particularly on the transcriptional profile of genes that moved into the *Ectocarpus* sp. SDR, because this is the species for which the highest quality male and female gametophyte transcriptomic data are available. Remarkably, all except one of the genes that were gained by the female or male SDR of *Ectocarpus* sp. were expressed exclusively in the gametophyte generation (Additional file 2: Figure S3), suggesting a sex-specific role in reproduction. Interestingly, the genes that are located on the PAR of *Ectocarpus* sp. but that have moved into the SDR of the kelps exhibited either a gametophyte-biased (Ec-13_001410) or a male-biased (Ec-13_002450) expression pattern in *Ectocarpus* sp. (Additional file 2: Figure S3).

### Mechanism of gene relocation in *S. japonica*

Gene transposition may occur through gene duplication, either by a DNA-based mechanism (ectopic recombination) or by an RNA-based mechanism (retrotransposition by reverse transcription of an mRNA). Once a duplicate has arisen in a new location, the original copy can be either maintained or lost. We refer to the former case as duplicative transpositions and the latter as relocations, following [44].

At least three genes moved out of the ancestral SDR by a DNA-based mechanism in the lineage leading to *S. japonica* (SJ15874, SJ05597, SJ18945). The original, sex-linked copy was presumably lost and all the three autosomal duplicated copies were functional (i.e. expressed during the life cycle; Additional file 1: Table S4).

We detected three cases of retrotranspositions from the SDR to the autosomes in the lineage leading to *S. japonica*. These three *S. japonica* genes (SJ13722, SJ09422 and SJ15797) shared between 51 and 81% similarity with their *Ectocarpus* SDR homologues at the protein level. Two of these genes (SJ13722, SJ09422) contained no introns, whereas one (SJ15797) appears to have gained one additional exon and intron either during the retrotransposition process or subsequent to it (Fig. 4a; Additional file 1: Table S7). SJ09422 was truncated and appeared to be non-functional and was therefore classified as a pseudo-retrogene. We could not find the progenitor copy of any of these retrogenes in the *S. japonica* genome, indicating that they are orphan retrogenes, i.e. retrotransposed genes where the progenitor copy has been lost (relocations) [45].

To assess the abundance of orphan retrogenes genome-wide in *S. japonica*, we searched for functional, intronless genes that matched intron-rich homologues in *Ectocarpus* sp. (see “Methods” for details). This screen detected a total of 16 orphan retrogenes, all of which were located on autosomes. Note that this set of 16 orphan retrogenes included SJ13722 but neither SJ15797 nor SJ09422 because strict criteria were required to clearly discriminate retrogenes from other types of duplicated loci in this genome-wide screen. These strict criteria eliminated candidates that contained introns (e.g. SJ15797) and truncated, non-functional loci (e.g. SJ09422). We then used the genomic locations of the *Ectocarpus* sp. homologues of the 16 *S. japonica* orphan retrogenes to infer the probable genomic location of the parental kelp genes that gave rise to these retrogenes. Strikingly, the *Ectocarpus* homologues of six of the 16 *S. japonica* orphan retrogenes were located on the *Ectocarpus* sex chromosome (Additional file 1: Table S7), suggesting that *S. japonica* orphan retrogenes have arisen preferentially from genes located on the ancestral sex chromosome. Moreover, the sex chromosome-derived retrogenes exhibited significantly higher expression levels than retrogenes that were derived from autosomal loci (Wilcoxon test, *p* = 0.01934; Fig. 4b), suggesting that retrotransposition events originating from the sex chromosome are more likely to generate functional retrogenes.

## Discussion

### Ectocarpales and kelp sex chromosomes are derived from a common ancestral sex chromosome and share a candidate sex-determining gene

The presence of shared orthologues in the SDRs of the Ectocarpales and kelps strongly suggests that the U and V chromosomes are derived from the same ancestral autosome, which therefore originated at least 80–110 MY ago. This is consistent with previous work which also suggested that these brown algal U and V chromosomes are evolutionarily old [27, 28]. An earlier study indicated that a locus on the V actively determines male sex, whereas expression of the female sex may just require absence of the V chromosome (i.e. be the default sex) [28]. The HMG domain gene Ec-13_001750 was proposed as a strong candidate for the male sex-determining gene. This suggestion is supported by the observation, in this study, that all the brown algae investigated possessed orthologues of Ec-13_001750 in their male SDRs.

### Empirical support for theoretical predictions on UV evolution

It is a long-standing prediction that only genes implicated in roles shared by both sexes will be maintained as functional copies in both U- and V-specific regions, while genes with only male or only female functions can be gained specifically in the sex they are needed for or be lost from the chromosome type that does not occur in that sex [21, 23]. More recent models also predict that one member of a gametologue pair will degenerate in UV systems if the genes are required only during the diploid phase of the life cycle [21]. Our data provide empirical support for these theoretical predictions. *Ectocarpus* genes that have functional copies in both the U and V regions (gametologues) were expressed at similar levels in males and females and ubiquitously throughout the life cycle, indicating they have functions that are shared by both male and female individuals. In contrast, sex-limited genes exhibited gametophyte-specific expression patterns, suggesting that they have a function during the sexual phase of the life cycle. Interestingly, the vast majority of the sex-limited genes have been gained by the SDR in a lineage-specific fashion (from the PAR or from autosomal locations) and seem to have acquired sex-specific roles since they moved into the SDR. Loss of genes from only one of the haplotypes (U or V) seems to be less frequent in the species studied here, and is consistent with the globally low level of SDR degeneration in *Ectocarpus* [28]. Some cases of loss of both gametologues were, however, observed in the kelps, and in these cases the autosomal paralog may have compensated functionally for the loss of the SDR genes.

The consequences of sex linkage for the molecular evolution of genes seems to be similar for the different brown algal species and can probably be attributed to less efficient selection in non-recombining regions as a result of Hill-Robertson interference [30]. The faster evolutionary rates of the SDR genes could also be a result of positive selection, but we did not find evidence of adaptive evolution for any of the genes or species studied here.

### Genes move into the SDR by two different mechanisms

Despite their shared ancestry, brown algal U and V SDR haplotypes contain a proportion of young, lineage-specific genes such as the four genes that are predicted to have moved into the SDR during the divergence of the genus *Ectocarpus* (Ec-13_001930, Ec-13_002030, Ec-sdr_f_000050 and Ec-sdr_f_000120; Fig. 1). The genus *Ectocarpus* is estimated to have diverged from its sister genus, *Kuckuckia*, between 12 and 27 MY ago [46], indicating that these lineage-specific acquisitions occurred within the previous 27 MY. The evolution of this UV system has therefore been driven by gene gain and gene loss, for both the U- and the V-specific regions (Fig. 2). We identified two mechanisms of gene movement into the SDR. One involved movement of genes from an unlinked chromosomal location to the SDR, presumably by a process of duplication and transposition (this was seen, for instance, in the case of genes moving into the *Ectocarpus* sp. female SDR). The second process appears to involve engulfment of genes located on the PAR by an expanding SDR, and this was observed in the kelp lineage. Note that the second process can occur without the gene needing to be moved out of its local syntenic context, although some degree of chromosomal rearrangement may be involved, such as inversion events for example.

### An excess of UV-derived orphan retrogenes in *S. japonica*

Gene movement out of the sex chromosome appears to have involved both DNA-based (ectopic recombination) and RNA-based (retrotransposition) mechanisms. Comparative analysis of the genomes of *S. japonica* and *Ectocarpus* sp. identified 16 orphan retrogenes in *S. japonica*. Recent studies have revealed that retrocopies often replace their parental genes in animal and green lineage genomes [45, 47, 48]. The results presented here indicate that this phenomenon may have a significance in additional eukaryotic supergroups, in this case the stramenopiles.

Strikingly, in the lineage leading to *S. japonica*, UV sex chromosomes appear to have generated a disproportional amount of functional orphan retrogenes compared to the autosomes. This difference could be linked to the increased tendency for sex chromosome genes to degenerate, particularly if they are located in the non-recombining SDR. The survival of a sex chromosome-derived retrogene would therefore be a potential mechanism to maintain the function of the parent gene in the genome. Consistent with this hypothesis, one of the three female gametologue pseudogenes in the *Ectocarpus* sp. SDR has a functional, retrotransposed homologue in the autosomes of *S. japonica*. This ‘rescue’ system can be equated to the recently proposed mechanism for relocation of Y genes in mammals, where Y-linked gene loss is a driver of gene transposition from the Y towards the autosomes [49].

## Conclusions

This study affords the first analysis of the dynamics of gene traffic in a haploid UV sexual system and identifies several features of brown algal sex chromosomes that may be of general relevance to the evolution of this poorly studied class of sex-determining system. Although a core set of genes has been maintained in the brown algal SDRs over the last 80–110 MY, both gene gain and gene loss have played an important role in shaping the evolution of these chromosomal regions. Gene gain by the U and V SDRs has involved both duplication and transposition from other chromosomes and engulfment of neighbouring genes located on the PAR. Moreover, consistent with theoretical models on sex chromosome evolution, expression analysis indicated that the genes that have entered the U- or the V-specific regions have a role during the reproduction of the corresponding sex. Although the SDR has been relatively well preserved from degeneration by haploid purifying selection, expression analysis indicated that genes that are only required during the diploid generation tend to have been lost. Genes have also moved off the sex chromosome, either by direct transposition or by retrotransposition. Duplicative movement of genes off the U/V sex chromosomes to the autosomes could provide a mechanism to maintain these genes in the genome if they are subsequently lost from the SDR as a result of degeneration of this region. Brown algal SDR genes were found to be evolving rapidly, and this has also been observed in XY and ZW systems. However, whilst there is evidence that positive selection drives the rapid evolution of a subset of Y/W genes in the latter, we found no evidence for this phenomenon in the brown algal UV system. It will be of great interest in the future to determine whether these various observations represent general features of UV sexual systems or are specific to the brown algae.

Finally, comparative analysis of brown algal SDRs showed that only one sex-limited SDR gene, a HMG domain protein that had previously been proposed as a candidate sex-determining gene, was conserved in all the species analysed. If future analyses confirm that this gene determines sex in these species, this will represent an important step towards understanding haploid sex determination at the mechanistic level.

## Methods

### Biological material and generation of genomic and transcriptomic sequence data

The strains used in this study are listed in Additional file 1: Table S2. We sequenced genomic DNA from male and female haploid gametophytes of *E. siliculosus*, *E. fasciculatus*, *S. lomentaria* and *M. pyrifera* using Illumina HiSeq 2000 paired-end technology with a read length of 125 bp (Fasteris, Switzerland). RNA-seq data for *M. pyrifera* male and female gametophytes (triplicates) and *S. lomentaria* male and female gametophytes (duplicates) were generated using Illumina HiSeq 2000 paired-end technology with a read length of 125 bp (Fasteris, Switzerland). Accession numbers for all sequence data are given in Additional file 1: Table S2.

### Genome and transcriptome assemblies

Read quality was assessed with FastQC (http://www.bioinformatics.babraham.ac.uk/projects/fastqc), and low quality bases and adapter sequences were trimmed using Trimmomatic (leading and trailing bases with quality below 3 and the first 12 bases were removed, minimum read length 50 bp) [50]. High score reads (pooled from males and females) were assembled using SOAPdenovo2 [35] with an appropriate kmer value chosen using Kmergenie [51]. *E. siliculosus* male and female genomic data were aligned to the reference genome (*Ectocarpus* sp.), and consensus sequences of coding regions with at least 10× coverage were recovered using the CLC Assembly Cell (www.clcbio.com). Transcriptome assemblies were generated using the Trinity *de novo* assembler [52] with default parameters and normalised mode. Assembly statistics were performed with Abyss [53].

### Identification of sex-linked scaffolds

SDR sequences were identified based on differences in read coverage of de novo assembled scaffolds in males versus females. First, the sequencing reads from both sexes were mapped onto the de novo assembled reference genomes using Bowtie2 [54] and filtered for mapping quality (MQ ≥20). Next, the coverage of each scaffold was calculated as number of mapped reads normalised for scaffold length (reads per kilobase). Scaffolds with a depth of coverage in the range of the genome average for only one sex and markedly lower or no coverage for the other sex were identified as potentially sex-linked. To detect candidate sex-linked scaffolds, *Ectocarpus* sp. genes were blasted (tblastn, E-value cutoff 10e^–4^) against the *E. fasciculatus*, *M. pyrifera*, S. *lomentaria*, *U. pinnatifida* and S. *japonica* genomic scaffolds. The best matching sequences were blasted back to the *Ectocarpus* sp. proteins (blastx, E-value cutoff 10e^–4^) and were retained if they harboured an ortholog of an *Ectocarpus* sp. sex-linked gene and if they met the coverage criteria (Additional file 1: Table S8). *E. siliculosus* male and female SDR sequences were obtained from the guided genome assembly described above.

RNA-seq data from males and females of *U. pinnatifida* [55], *M. pyrifera* and *S. lomentaria* were mapped to the de novo assembled genomes. Mapping of reads in a sex-specific manner to previously identified sex-linked scaffolds further confirmed sex linkage. Sequences of the sex-linked genes for which there were no de novo transcripts or annotated gene model (*S. japonica*) were recovered from identified sex scaffolds using Fgenesh + protein-based gene prediction [56] and the corresponding *Ectocarpus* protein as a reference [57]. The published *S. japonica* genome represents only one sex. We therefore used DNA-seq data from four heterozygous diploid sporophytes and two gametophytes in combination with genomic information from the deep sequenced *S. japonica* reference strain to identify scaffolds corresponding to the other sex [29] (Additional file 1: Table S2). Our reasoning was that sequences present in the sporophyte data (where both U and V are present) but absent from either male (V) or female (U) gametophyte data would correspond to the SDR sequences of the opposite sex (Additional file 2: Figure S4). The Illumina reads from the four sporophytes were aligned to the available *S. japonica* genome using Bowtie2 [54]. The pool of reads that did not match the reference genome sequence were subtracted using seqtk (https://github.com/lh3/seqtk) and used for de novo assembly with SOAPdenovo2 [58]. Next, the DNA-seq reads from the four *S. japonica* sporophytes, as well as reads from the two gametophytes, were mapped to the reference genome and to the *de novo* assembled putative sex-specific scaffolds using Bowtie2 and filtered for mapping quality (MQ ≥20). Coverage of each scaffold was calculated using HTSeq [59] and normalised for scaffold length (reads per kilobase). We then searched for scaffolds with 30–70% of the average coverage with at least three of the four sporophyte samples and average or no coverage with gametophyte data. The reasoning was that the diploid sporophytes will contain each autosome in two copies but only one copy of each sex chromosome (U and V); therefore, the depth of coverage for an autosomal sequence should be twice that for sex-specific sequence. On the other hand, depth of coverage for U-linked or V-linked scaffolds in a haploid gametophyte would be the same as for an average autosome. This approach identified 75 putative sex-linked scaffolds, several of which matched *Ectocarpus* sp. SDR (and PAR) genes (blastx, cutoff 10e^–4^), providing further support for sex linkage. Interestingly, although the sequenced reference strain was described as a female [29], the scaffolds of this assembly matched only male-specific genes, suggesting that it in fact corresponds to a male.

To confirm sex linkage of the putative sex-linked scaffolds and determine the sex of the reference strain, we selected five male and five female *S. japonica* gametophytes and cultivated them to sexual maturity (Additional file 1: Table S9). DNA was extracted separately from individuals producing either oogonia (females) or antheridia (males) (Additional file 2: Figure S4b) using the NucleoSpin Plant II kit (Macherey-Nagel) and was amplified with PCR primers specific to each putative sex-linked scaffold (Additional file 1: Table S10). Sixteen scaffolds that were assembled de novo from reads that did not match the reference genome (cumulative size of 50 kbp) were found to be female-specific. In addition, primer pairs corresponding to 59 scaffolds from the reference genome sequence (cumulative size 4.91 Mbp) amplified products only from male DNA. Based on these PCR tests, together with the blast and depth of coverage results, we concluded that the genome sequenced strain of *S. japonica* described in [29] carried the V, and not the U, sex chromosome (Additional file 1: Table S11). Throughout, we therefore considered that the reference *S. japonica* strain was a male.

We describe genes as being lost if we failed to identify an orthologous sequence in assembled genomes of the target species using a Blastp cutoff of <10e^–04^. This approach may have failed to detect very highly divergent orthologues but we considered that such a high degree of divergence would unlikely be consistent with conservation of gene function.

### Alignments and phylogenetic trees

All sequence alignments were done with Tcoffee [60] and curated with Gblocks [61]. Maximum likelihood gene trees were based on protein alignments and performed using phylogeny.fr [62] with 500 bootstraps. Alignments shorter than 100 bp were discarded.

### Molecular evolution analysis

We investigated the evolutionary consequences of sex-linkage by comparing cross-species synonymous and nonsynonymous divergence between *Ectocarpus* sp. and *E. fasciculatus*. The divergence time between the two *Ectocarpus* species is large enough to infer patterns of evolution in coding genes but is small enough to allow high confidence alignments of orthologues at the nucleotide level.

We estimated pairwise dN and dS using the Phylogenetic Analysis by Maximum Likelihood (PAML4) algorithm (CODEML, F3x4 model, runmode = −2) [63]. Calculation of divergence between gametologue pairs within each species was performed in the same manner. Codon usage bias (analysed as CAI) was calculated using the CAIcal server (http://genomes.urv.es/CAIcal/) [64]. Divergence and CAI values for the sex-biased and unbiased genes were published earlier [33].

### Positive selection analysis

We used the sequence data from the three *Ectocarpus* species and *S. lomentaria* to search for evidence of positive selection. Curated protein alignments of sex-linked genes were submitted to Pal2Nal to recover the corresponding alignment in nucleotides. Levels of nonsynonymous (dN) and synonymous (dS) substitution were estimated by the maximum-likelihood method available in the CODEML program (PAML4 package) using the F3x4 model of codon frequencies and a user tree specified according to the phylogeny of cox3 marker (Additional file 2: Figure S1). CODEML paired nested site models (M0, M1a, M2a; M7, M8) of sequence evolution were used and the outputs compared using the likelihood ratio test. Empirical Bayes methods allowed for identification of positively selected sites a posteriori.

### Gene expression analysis

The transcriptome data and gene expression data for *Ectocarpus* sp. across different life stages have been described previously [33]. RNA-seq data for *S. japonica* gametophytes, spores and two sporophytes were obtained from the Sequence Read Archive and GEO database at NCBI (Additional file 1: Table S2). Mapping to the reference genome (*S. japonica*) or reference transcriptome (*M. pyrifera*) was done using TopHat2 with the Bowtie2 aligner [54]. The mapped sequencing data were then processed with HTSeq [59] and used to calculate expression values as TPM (transcripts per million). Only genes with TPM >1 were considered to be expressed.

All statistical analyses were performed in R suite v3.3.0.

### Identification of retrogenes

Two approaches were used to identify retrogenes in *Ectocarpus* sp. and *S. japonica*. First, the database of all *Ectocarpus* sp. predicted protein sequences was mapped against itself using Blastp with a threshold E-value of 0.001, and similarly the database of all *S. japonica* predicted protein sequences [29] was mapped against itself and matching pairs of genes retained. In the second approach, a combined dataset containing all the *Ectocarpus* sp. predicted protein sequences plus all the *S. japonica* predicted protein sequences was mapped against itself in order to identify orphan retrogenes in each species that had lost the parental copy of the gene in that species but still maintained the parental copy in the other species. Retrogene candidates were identified from among the matching gene pairs of both approaches on the following criteria: (1) Genes had to align over at least 70% of the length (for both genes) with a minimum alignment length of 35 amino acids and share at least 50% amino acid identity. (2) The retrogene candidate had to be mono-exonic and the parental gene to contain at least two introns. Criteria 1 and 2 were necessary because of the presence of a large number of partial, DNA-based duplications where intron loss was not due to retrotransposition, as well as to the presence of large gene families. (3) A single best parent could be identified in the data set with a difference of at least 5% amino acid identity compared to the next most similar gene in the genome. This criterion was necessary to exclude gene families with an unclear evolutionary relationship and transposable elements that were incorrectly annotated as genes. Moreover, in the cross-species search for orphan retrocopies this requirement establishes that a retrogene present in one species with a homologue of its parent present in the other species does not contain an equally closely related homologue within the first species. (4) Candidates were manually inspected to verify the loss of introns in the candidate retrogene. Note that this method for identifying retrogenes will not retrieve all potential retrogenes. This method relies on annotated genes and thus unannotated pseudoretrogenes cannot be identified. The stringent criteria for intron loss also exclude genes that have potentially gained introns since the retrotransposition event and retrogenes that have integrated into other genes as exons.

The first method identified four and two potential retrogene candidates that had maintained their parental copy in *Ectocarpus* sp. and S. *japonica*, respectively. The small number of duplicative retrogene candidates identified using this approach indicates that such retrotransposition events are rare, that there is selection against maintenance of retrogenes (and/or against maintenance of the progenitor copy after retrotransposition), or that retrogenes are unidentifiable because they rapidly gain introns.

## Additional files

Additional file 1: Supplementary Tables. **Table S1**. Genes present in the male and female sex-specific region of the *Ectocarpus* sp. sex chromosome. **Table S2.** Strains used in this study, sequencing statistics and accession numbers. **Table S3.** Synonymous nucleotide substitutions (dS) for gametologue pairs in the brown algae included in this study. **Table S4.** Molecular evolutionary features and transcript levels of *Ectocarpus* sp. and *S. japonica* sex-linked genes. **Table S5.** PAML codeml analysis for evidence of positive selection acting on SDR genes using sequences from three *Ectocarpus* species and *S. lomentaria*. **Table S6.** Sex-linked genes and their autosomal homologues in *Ectocarpus* sp. (*Esp*), *S. lomentaria* (*Slom*), *S. japonica* (*Sjap*), *M. pyrifera* (*Mpyr*) and *U. pinnatifida* (*Upin*). **Table S7.** Orphan retrogenes in *S. japonica* for which the parental copy homologue is still present in *Ectocarpus* sp. genome. **Table S8.** Nucleotide and protein sequence of the orthologs of the *Ectocarpus* sp. male and female SDR genes recovered from *E. siliculo*sus, *E. fasciculatus*, *S. lomentaria*, *S. japonica*, *M. pyrifera* and *U. pinnatifida.* **Table S9.** Strains of *S. japonica* used in this study to correlate phenotypic and genotypic sex. **Table S10.** Primers used to determine sex linkage in *S. japonica*. **Table S11** Assessment of phenotypic and genotypic sex in *S. japonica* individuals. (XLSX 318 kb)

Additional file 2: Supplementary Figures. **Figure S1.** Maximum likelihood phylogenetic trees of protein sequences of U-linked, V-linked and autosomal homologues across the seven species of brown algae. **Figure S2.** Patterns of expression of three *Ectocarpus* sp. male-limited SDR genes during the life cycle. **Figure S3.** Abundance of *Ectocarpus* sp. SDR gene transcripts during the gametophyte and sporophyte generations of the life cycle. **Figure S4.** The life cycle of the kelp *S. japonica*. (DOCX 9962 kb)

## References

[1] Betran E, Thornton K, Long M (2002). Retroposed new genes out of the X in Drosophila. Genome Res.

[2] Emerson JJ, Kaessmann H, Betran E, Long M (2004). Extensive gene traffic on the mammalian X chromosome. Science.

[3] Potrzebowski L, Vinckenbosch N, Marques AC, Chalmel F, Jegou B, Kaessmann H (2008). Chromosomal gene movements reflect the recent origin and biology of therian sex chromosomes. PLoS Biol.

[4] Sturgill D, Zhang Y, Parisi M, Oliver B (2007). Demasculinization of X chromosomes in the Drosophila genus. Nature.

[5] Vibranovski MD, Koerich LB, Carvalho AB (2008). Two new Y-linked genes in Drosophila melanogaster. Genetics.

[6] Bergero R, Qiu S, Charlesworth D (2015). Gene loss from a plant sex chromosome system. Curr Biol.

[7] Charlesworth B, Coyne JA, Barton NH (1987). The relative rates of evolution of sex chromosomes and autosomes. Am Nat.

[8] Bachtrog D, Toda NRT, Lockton S (2010). Dosage compensation and demasculinization of X chromosomes in Drosophila. Curr Biol.

[9] Vicoso B, Charlesworth B (2009). Effective population size and the faster-X effect: an extended model. Evol Int J Org Evol.

[10] Tao Y, Araripe L, Kingan SB, Ke Y, Xiao H, Hartl DL (2007). A sex-ratio meiotic drive system in Drosophila simulans. II: An X-linked distorter. PLoS Biol.

[11] Wang J, Long M, Vibranovski MD (2012). Retrogenes moved out of the z chromosome in the silkworm. J Mol Evol.

[12] Bellott DW, Skaletsky H, Pyntikova T, Mardis ER, Graves T, Kremitzki C (2010). Convergent evolution of chicken Z and human X chromosomes by expansion and gene acquisition. Nature.

[13] Bachtrog D (2013). Y chromosome evolution: emerging insights into processes of Y chromosome degeneration. Nat Rev Genet.

[14] Smeds L, Warmuth V, Bolivar P, Uebbing S, Burri R, Suh A (2015). Evolutionary analysis of the female-specific avian W chromosome. Nat Commun.

[15] Koerich LB, Wang X, Clark AG, Carvalho AB (2008). Low conservation of gene content in the Drosophila Y chromosome. Nature.

[16] Carvalho AB, Lazzaro BP, Clark AG (2000). Y chromosomal fertility factors kl-2 and kl-3 of Drosophila melanogaster encode dynein heavy chain polypeptides. Proc Natl Acad Sci U S A.

[17] Carvalho AB, Vicoso B, Russo CAM, Swenor B, Clark AG (2015). Birth of a new gene on the Y chromosome of Drosophila melanogaster. Proc Natl Acad Sci U S A.

[18] Ishizaki K, Shimizu-Ueda Y, Okada S, Yamamoto M, Fujisawa M, Yamato KT (2002). Multicopy genes uniquely amplified in the Y chromosome-specific repeats of the liverwort Marchantia polymorpha. Nucleic Acids Res.

[19] Matsunaga S, Isono E, Kejnovsky E, Vyskot B, Dolezel J, Kawano S (2003). Duplicative transfer of a MADS box gene to a plant Y chromosome. Mol Biol Evol.

[20] Bachtrog D, Mank JE, Peichel CL, Kirkpatrick M, Otto SP, Ashman T-L (2014). Sex determination: why so many ways of doing it?. PLoS Biol.

[21] Immler S, Otto SP (2015). The evolution of sex chromosomes in organisms with separate haploid sexes. Evolution.

[22] Charlesworth B, Charlesworth D (2000). The degeneration of Y chromosomes. Philos Trans R Soc Lond B Biol Sci.

[23] Bull JJ (1978). Sex chromosomes in haploid dioecy: a unique contrast to Muller’s theory for diploid dioecy. Am Nat.

[24] Kawai H, Hanyuda T, Draisma SGA, Wilce RT, Andersen RA (2015). Molecular phylogeny of two unusual brown algae, Phaeostrophion irregulare and Platysiphon glacialis, proposal of the Stschapoviales ord. nov. and Platysiphonaceae fam. nov., and a re-examination of divergence times for brown algal orders. J Phycol.

[25] Silberfeld T, Leigh JW, Verbruggen H, Cruaud C, de Reviers B, Rousseau F (2010). A multi-locus time-calibrated phylogeny of the brown algae (Heterokonta, Ochrophyta, Phaeophyceae): Investigating the evolutionary nature of the “brown algal crown radiation”. Mol Phylogenet Evol.

[26] Luthringer R, Cormier A, Peters AF, Cock JM, Coelho SM (2015). Sexual dimorphism in the brown algae. Perspectives Phycol.

[27] Lipinska AP, Ahmed S, Peters AF, Faugeron S, Cock JM, Coelho SM (2015). Development of PCR‐based markers to determine the sex of kelps. PLoS ONE.

[28] Ahmed S, Cock JM, Pessia E, Luthringer R, Cormier A, Robuchon M (2014). A haploid system of sex determination in the brown alga Ectocarpus sp. Curr Biol.

[29] Ye N, Zhang X, Miao M, Fan X, Zheng Y, Xu D (2015). Saccharina genomes provide novel insight into kelp biology. Nat Commun.

[30] Campos JL, Charlesworth B, Haddrill PR (2012). Molecular evolution in nonrecombining regions of the Drosophila melanogaster genome. Genome Biol Evol.

[31] Pal C, Papp B, Hurst LD (2001). Highly expressed genes in yeast evolve slowly. Genetics.

[32] Subramanian S, Kumar S (2004). Gene expression intensity shapes evolutionary rates of the proteins encoded by the vertebrate genome. Genetics.

[33] Lipinska A, Cormier A, Luthringer R, Peters AF, Corre E, Gachon CMM (2015). Sexual dimorphism and the evolution of sex-biased gene expression in the brown alga ectocarpus. Mol Biol Evol.

[34] Cortez D, Marin R, Toledo-Flores D, Froidevaux L, Liechti A, Waters PD (2014). Origins and functional evolution of Y chromosomes across mammals. Nature.

[35] Bachtrog D (2004). Evidence that positive selection drives Y-chromosome degeneration in Drosophila miranda. Nat Genet.

[36] Gerrard DT, Filatov DA (2005). Positive and negative selection on mammalian Y chromosomes. Mol Biol Evol.

[37] Larracuente AM, Clark AG (2013). Surprising differences in the variability of Y chromosomes in African and cosmopolitan populations of Drosophila melanogaster. Genetics.

[38] Galtier N, Piganeau G, Mouchiroud D, Duret L (2001). GC-content evolution in mammalian genomes: the biased gene conversion hypothesis. Genetics.

[39] Eyre-Walker A (1999). Evidence of selection on silent site base composition in mammals: potential implications for the evolution of isochores and junk DNA. Genetics.

[40] Luthringer R, Lipinska AP, Roze D, Cormier A, Macaisne N, Peters AF (2015). The pseudoautosomal regions of the U/V sex chromosomes of the brown alga Ectocarpus exhibit unusual features. Mol Biol Evol.

[41] Herpin A, Schartl M (2015). Plasticity of gene-regulatory networks controlling sex determination: of masters, slaves, usual suspects, newcomers, and usurpators. EMBO Rep.

[42] Li W, Sullivan TD, Walton E, Averette AF, Sakthikumar S, Cuomo CA (2013). Identification of the mating-type (MAT) locus that controls sexual reproduction of Blastomyces dermatitidis. Eukaryot Cell.

[43] Jordan CY, Charlesworth D (2012). The potential for sexually antagonistic polymorphism in different genome regions. Evol Int J Org Evol.

[44] Meisel RP (2009). Evolutionary dynamics of recently duplicated genes: Selective constraints on diverging paralogs in the Drosophila pseudoobscura genome. J Mol Evol.

[45] Ciomborowska J, Rosikiewicz W, Szklarczyk D, Makalowski W, Makalowska I (2013). “Orphan” retrogenes in the human genome. Mol Biol Evol.

[46] Kawai H, Hanyuda T, Bolton J, Anderson R (2016). Molecular phylogeny of Zeacarpa (Ralfsiales, Phaeophyceae) proposing a new family Zeacarpaceae and its transfer to Nemodermatales. J Phycol.

[47] Carelli FN, Hayakawa T, Go Y, Imai H, Warnefors M, Kaessmann H (2016). The life history of retrocopies illuminates the evolution of new mammalian genes. Genome Res.

[48] Jakalski M, Takeshita K, Deblieck M, Koyanagi KO, Makalowska I, Watanabe H (2016). Comparative genomic analysis of retrogene repertoire in two green algae Volvox carteri and Chlamydomonas reinhardtii. Biol Direct.

[49] Hughes JF, Skaletsky H, Koutseva N, Pyntikova T, Page DC (2015). Sex chromosome-to-autosome transposition events counter Y-chromosome gene loss in mammals. Genome Biol.

[50] Bolger AM, Lohse M, Usadel B (2014). Trimmomatic: a flexible trimmer for Illumina sequence data. Bioinformatics.

[51] Chikhi R, Medvedev P (2014). Informed and automated k-mer size selection for genome assembly. Bioinformatics.

[52] Grabherr MG, Haas BJ, Yassour M, Levin JZ, Thompson DA, Amit I (2011). Full-length transcriptome assembly from RNA-Seq data without a reference genome. Nat Biotechnol.

[53] Simpson JT, Wong K, Jackman SD, Schein JE, Jones SJM, Birol I (2009). ABySS: a parallel assembler for short read sequence data. Genome Res.

[54] Langmead B, Salzberg SL (2012). Fast gapped-read alignment with Bowtie 2. Nat Methods.

[55] Shan TF, Pang SJ, Li J, Li X (2015). De novo transcriptome analysis of the gametophyte of Undaria pinnatifida (Phaeophyceae). J Appl Phycol.

[56] Salamov AA, Solovyev VV (2000). Ab initio gene finding in Drosophila genomic DNA. Genome Res.

[57] Cock JM, Sterck L, Rouzé P, Scornet D, Allen AE, Amoutzias G (2010). The Ectocarpus genome and the independent evolution of multicellularity in brown algae. Nature.

[58] Luo R, Liu B, Xie Y, Li Z, Huang W, Yuan J (2012). SOAPdenovo2: an empirically improved memory-efficient short-read de novo assembler. GigaScience.

[59] Anders S, Pyl PT, Huber W (2015). HTSeq--a Python framework to work with high-throughput sequencing data. Bioinformatics.

[60] Notredame C, Higgins DG, Heringa J (2000). T-Coffee: a novel method for fast and accurate multiple sequence alignment. J Mol Biol.

[61] Castresana J (2000). Selection of conserved blocks from multiple alignments for their use in phylogenetic analysis. Mol Biol Evol.

[62] Dereeper A, Guignon V, Blanc G, Audic S, Buffet S, Chevenet F (2008). Phylogeny.fr: robust phylogenetic analysis for the non-specialist. Nucleic Acids Res.

[63] Yang Z (2007). PAML 4: phylogenetic analysis by maximum likelihood. Mol Biol Evol.

[64] Puigbo P, Bravo IG, Garcia-Vallve S (2008). CAIcal: a combined set of tools to assess codon usage adaptation. Biol Direct.

