# Supplementary material for: Multiple gene movements into and out of haploid sex chromosomes
Source: Genome Biol. 2017 Jun 8;18:104. doi: 10.1186/s13059-017-1201-7 (PMC5463336; doi:10.1186/s13059-017-1201-7)

# Additional File 2

**Figure S1**. Maximum likelihood phylogenetic trees of protein sequences (PhyML, WAG substitution model) of U-linked (pink), V-linked (blue) and autosomal (green) homologues across the seven species of brown algae. When the genomic location is unclear, the sequence is marked in grey. Numbers indicate bootstrap support (500 iterations). E.sp, *Ectocarpus* sp.; Efasc, *E. fasciculatus*; Esil, *E. siliculosus*; Slom, *S. lomentaria*; Sjap, *S. japonica*; Mpyr, *M. pyrifera;* Upin, *U. pinnatifida*.


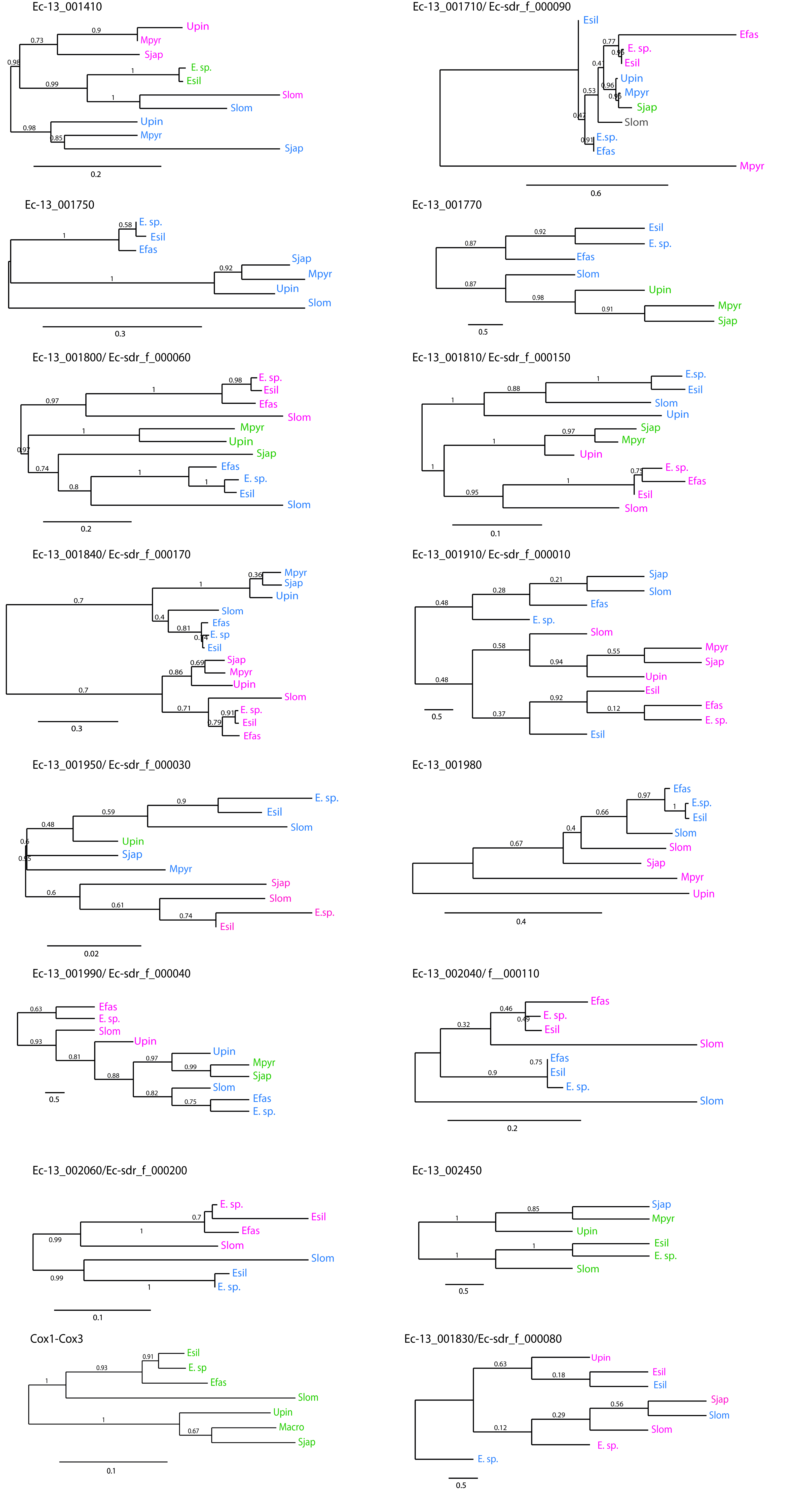


**Figure S2**. Patterns of expression of four *Ectocarpus* sp. male-limited SDR genes during the life cycle. immGA, immature gametophyte; matGA, mature gametophyte; SP, diploid sporophyte. Graphs represent means and standard error of the means. Different letters above the bars indicate statistically different datasets based on Welch Two Sample t-test (P<0.05).

**
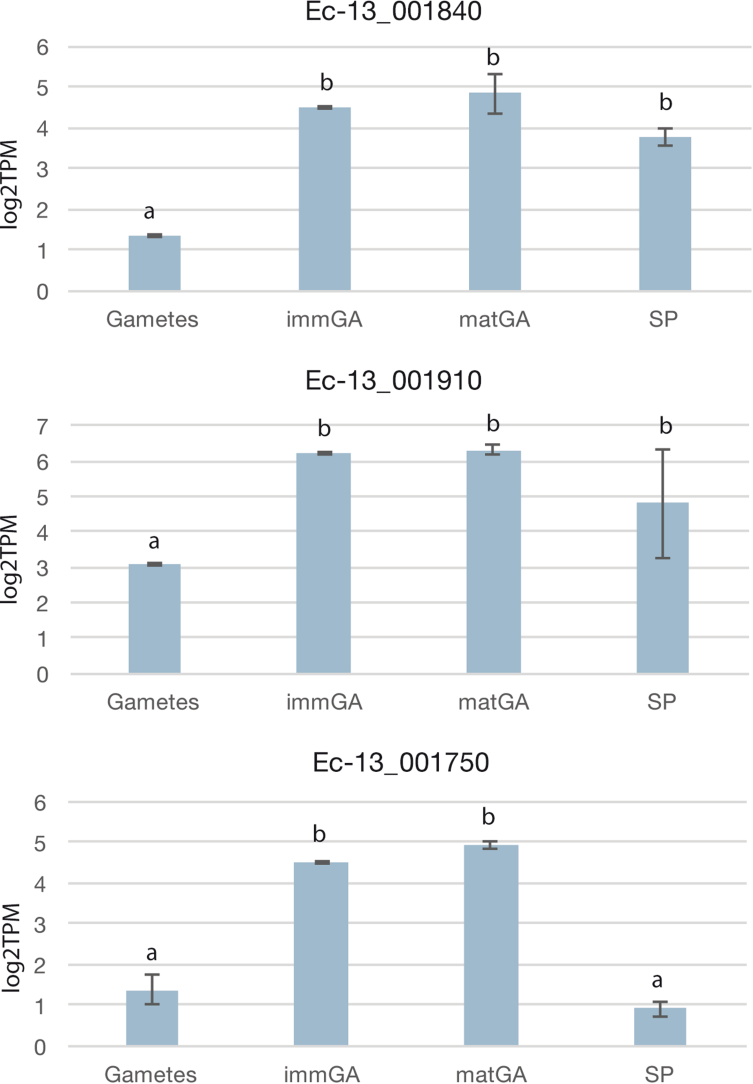
**

**Figure S3**. Abundance of *Ectocarpus* sp. SDR gene transcripts during the gametophyte and sporophyte generations of the life cycle. Genes that were absent in the ancestral SDR and entered the *Ectocarpus* SDR after divergence from the kelp lineage are marked in bold. Pseudogenes are represented in italics. Error bars represent standard errors of the mean. Asterisks highlight significant differences (Wilcox test), *P<0,05; **P<0,005; ***P<0,00005. Different letters indicate significant differences (Wilcox test, P<0,05). GA: gametophyte; SP: sporophyte.


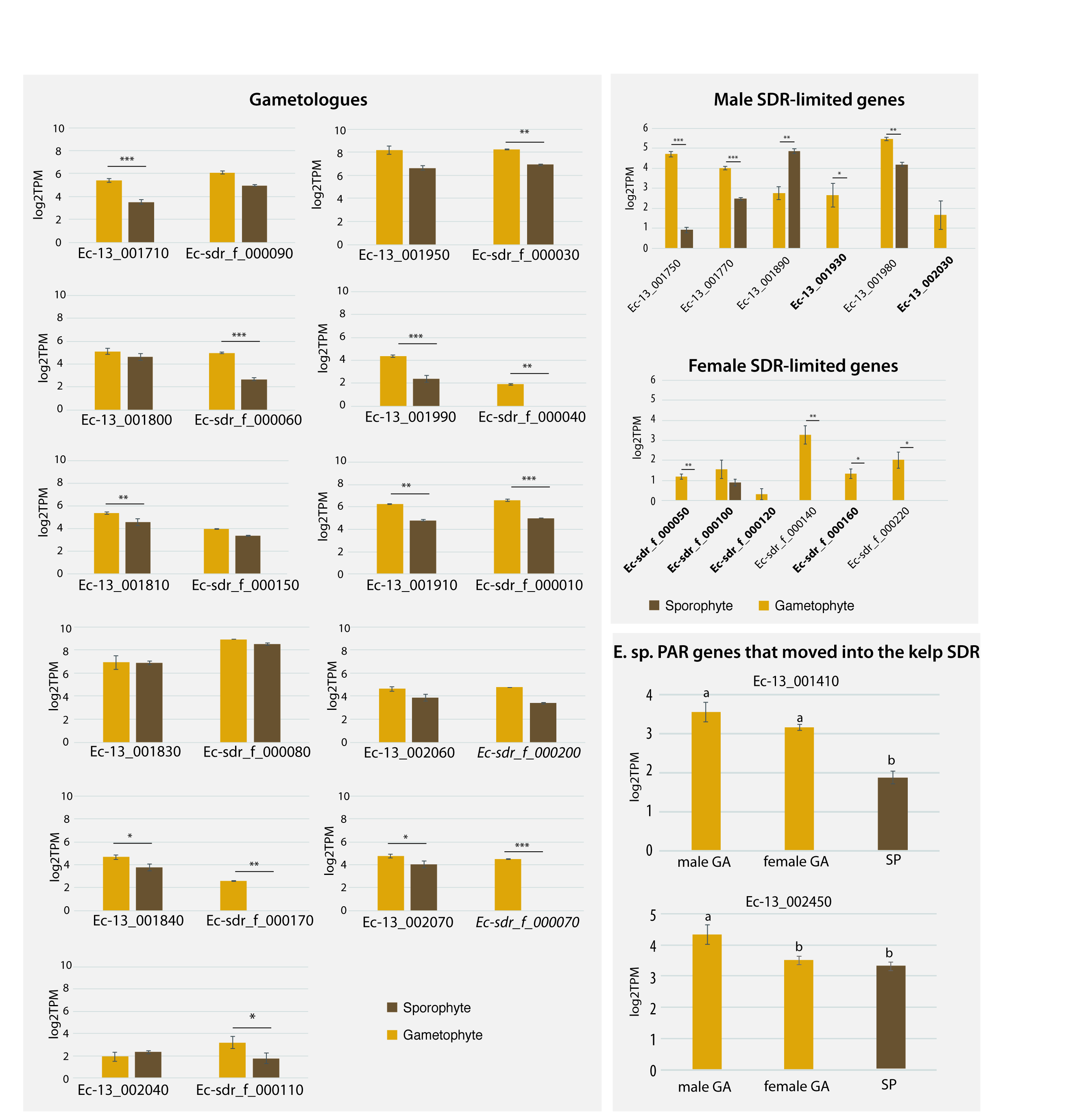


**Figure S4**. The life cycle of the kelp *S. japonica*. **A**) Haploid-diploid life cycle of *S. japonica*. Sex is determined after meiosis depending on whether the daughter cells inherit a V or a U chromosome. Meio-spores develop into multicellular, dimorphic gametophytes. The female and male gametophytes produce oogonia and antheridia where the eggs and sperm cells, respectively, are formed by mitosis. Fusion of gametes reconstitutes the diploid sporophyte. Both the complexity of the sporophyte generation compared to the gametophyte generation and the level of sexual dimorphism of the gametophytes are highly variable between species of brown alga. **B**) *S. japonica* female gametophytes. The asterisk indicates an oogonium, where female eggs are formed. **C**) *S. japonica* male gametophyte. The asterisk indicates an empty antheridium, from which sperm cells have been released. Note the smaller size of the male cells compared with those of the female gametophyte.


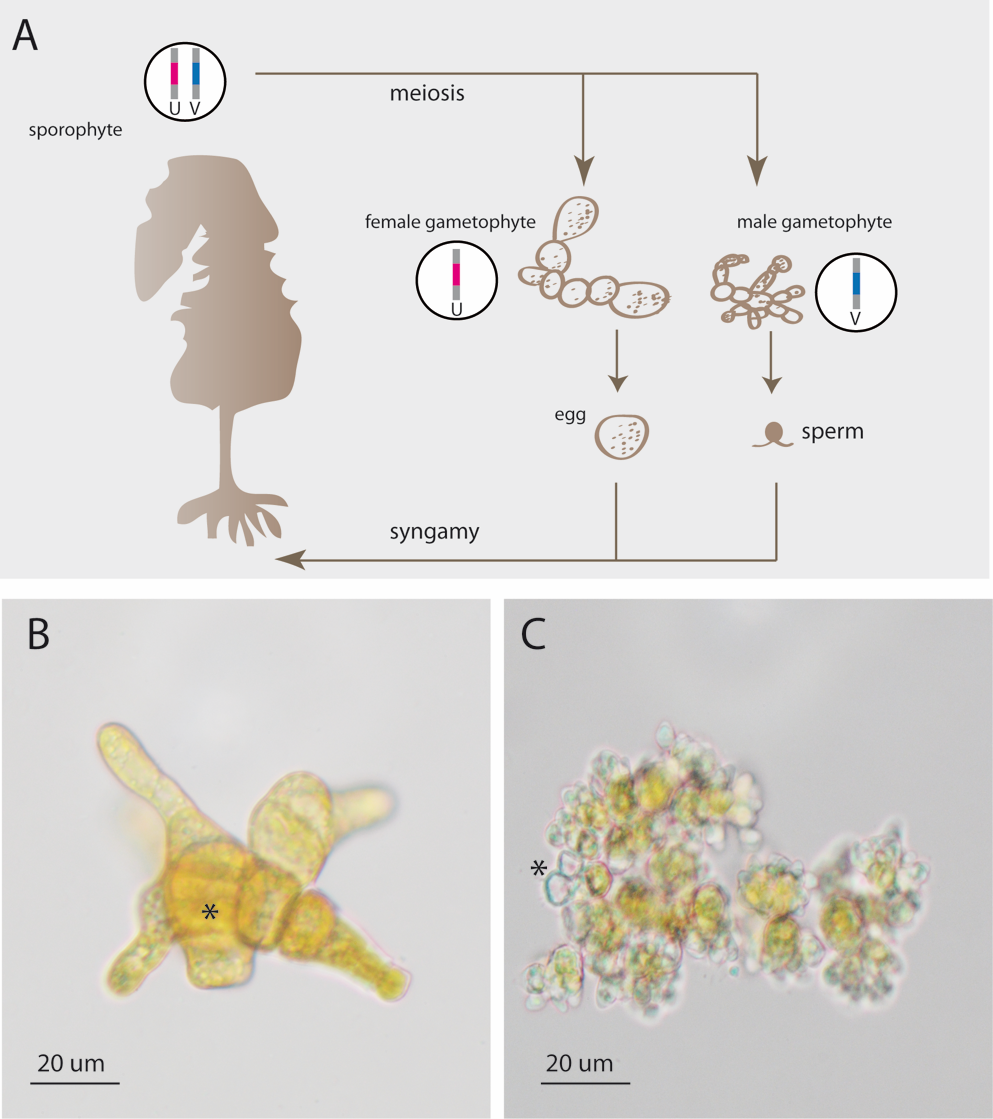

Supplement: Supplementary file 2 — Figure S1. Maximum likelihood phylogenetic trees of protein sequences of U-linked, V-linked and autosomal homologues across the seven species of brown algae. Figure S2. Patterns of expression of three Ectocarpus sp. male-limited SDR genes during the life cycle. Figure S3. Abundance of Ectocarpus sp. SDR gene transcripts during the gametophyte and sporophyte generations of the life cycle. Figure S4. The life cycle of the kelp S. japonica. (DOCX 9962 kb) [file 13059_2017_1201_MOESM2_ESM.docx]
